# A mutation in *POLE* predisposing to a multi-tumour phenotype

**DOI:** 10.3892/ijo.2014.2410

**Published:** 2014-04-29

**Authors:** ANNA ROHLIN, THEOFANIS ZAGORAS, STAFFAN NILSSON, ULF LUNDSTAM, JAN WAHLSTRÖM, LEIF HULTÉN, TOMMY MARTINSSON, GÖRAN B. KARLSSON, MARGARETA NORDLING

**Affiliations:** 1 Department of Clinical Genetics, Institute of Biomedicine, Sahlgrenska Academy at University of Gothenburg, Sahlgrenska University Hospital, SE 413 45 Gothenburg; 2 Mathematical Sciences, Chalmers University of Technology,SE 412 96 Gothenburg; 3 Department of Surgery, Institute of Clinical Sciences, Sahlgrenska Academy at University of Gothenburg; 4 The Colorectal Unit, Sahlgrenska Academy at University of Gothenburg, Sahlgrenska University Hospital/Östra, SE 416 85 Gothenburg; 5 The Swedish NMR-Centre, University of Gothenburg, SE 413 45 Gothenburg, Sweden

**Keywords:** colorectal cancer, mutation, POLE, exome sequencing

## Abstract

Somatic mutations in the *POLE* gene encoding the catalytic subunit of DNA polymerase ɛ have been found in sporadic colorectal cancers (CRCs) and are most likely of importance in tumour development and/or progression. Recently, families with dominantly inherited colorectal adenomas and colorectal cancer were shown to have a causative heterozygous germline mutation in the proofreading exonuclease domain of *POLE*. The highly penetrant mutation was associated with predisposition to CRC only and no extra-colonic tumours were observed. We have identified a mutation in a large family in which the carriers not only developed CRC, they also demonstrate a highly penetrant predisposition to extra-intestinal tumours such as ovarian, endometrial and brain tumours. The mutation, NM_006231.2:c.1089C>A, p.Asn363Lys, also located in the proofreading exonuclease domain is directly involved in DNA binding. Theoretical prediction of the amino acid substitution suggests a profound effect of the substrate binding capability and a more severe impairment of the catalytic activity compared to the previously reported germline mutation. A possible genotype to phenotype correlation for deleterious mutations in *POLE* might exist that needs to be considered in the follow-up of mutation carriers.

## Introduction

Two syndromes are associated with the major part of inherited colorectal cancer, hereditary non-polyposis colorectal cancer (HNPCC) and familial adenomatous polyposis (FAP). In Lynch syndrome families harbour a deleterious mutation in one of the mis-match repair (MMR) genes, *MLH1*, *MSH2*, *MSH6* or *PMS2*, and these families constitute 15–25% of families with HNPCC. In the major part of families with HNPCC no disease-causing mutations in known CRC predisposing genes can be found. Classical FAP is characterized by the development of multiple colorectal adenomas and is caused by mutations in the *APC* gene. It is today possible to detect nearly all causative mutations responsible for this syndrome. However, for the majority of patients who develop colorectal adenomas in the range from a few to approximately 100, the genetic basis of disease is yet to be unravelled. A limited number of these patients have attenuated FAP (AFAP) with less polyps and higher age at onset. In these cases the disease is caused by less severe mutations in the *APC* gene. Attenuated polyposis can also be caused by bi-allelic mutations in the *MUTYH* gene as seen in the recessively inherited MAP syndrome (*MUTYH*-associated polyposis).

Recently a new CRC syndrome, polymerase proofreading-associated polyposis was described (1). This syndrome is characterized by a dominantly inherited predisposition to the development of a variable number of colorectal adenomas and carcinomas. The proofreading (exonuclease) activity of the genes, *POLE* and *POLD1* (encoding the proofreading domain of polymerase ɛ and δ, respectively), are impaired by mutations in patients with this syndrome. The phenotypical expression of the disease varies; carriers of mutations in the *POLD1* gene developed endometrial tumours besides development of colorectal adenoma and carcinoma while the *POLE* mutation p.Leu424Val conferred a highly-penetrant predisposition only to colorectal adenoma and carcinoma in carriers.

We have identified a family with an amino-acid substitution p.Asn363Lys in *POLE*, in the same exonuclease domain of polymerase ɛ as the p.Leu424Val substitution. In this family mutation carriers are affected by a broad spectrum of tumours in addition to CRC. The substitution has a drastic affect on the proofreading capacity of the polymerase and we therefore suggest that a genotype to phenotype correlation might exist for this gene.

## Materials and methods

### Patients

All patients have given their consent and the study has been approved by the local ethics committee at the University of Gothenburg, Sweden.

**Figure 1.**
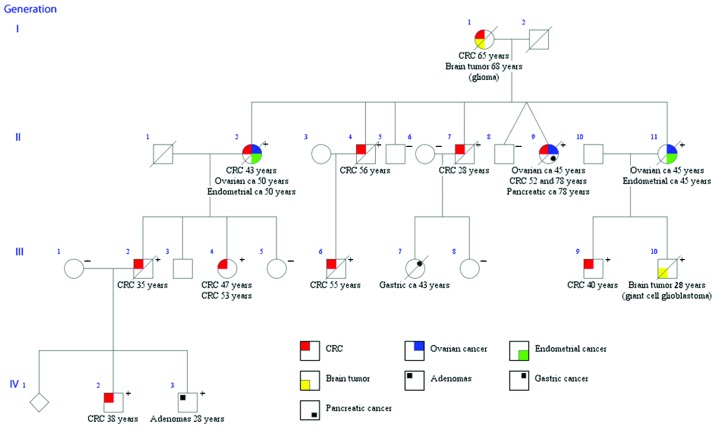
Pedigree of the family with the *POLE* c.1089C>A, p.Asn363Lys mutation. Whole-exome sequencing was performed on four individuals (III:1, III:4, IV:2 and IV:3). The mutation was present in heterozygous form in family members indicated by a plus (+). Family members negative for the mutation are indicated by a minus (−).

Family member III:4 (Fig. 1) had a prophylactic colectomy with ileo-rectal anastomosis (IRA) at 47 years with an accidental unexpected finding of CRC. She also developed a malign rectal tumour at 53 years. Two affected individuals in the family, I:1 and III:10 developed primary brain tumours, a glioma in one case and a giant cell glioblastoma in the other. Six family members >50 years of age, without sign of disease did not carry the mutation. Two of these individuals had normal barium enema at 59 (II:6) and 54 years (II:8) and they both reached an age of 84 years without any sign of the disease. Family member III:8 had normal colonoscopy at 46 years and member III:5 had prophylactic colectomy and IRA at 49 years and two normal rectoscopies after that at 54 and 58 years. Unfortunately family member III:7 with gastric cancer could not be tested due to no available material. Mutation testing was carried out by Sanger sequencing and/or TaqMan analyses on blood samples in most cases but for some individuals only paraffin-embedded tissue were available.

### DNA extraction and mutation screening

Genomic DNA was extracted using the BioRobot EZ1 (Qiagen, Hilden, Germany) with the EZ1 DNA Blood 350 *μ*l kit (Qiagen). DNA from adenomas, tumours and paraffin embedded tissues were extracted using QiAmp DNA microkit (Qiagen) following the manufacturer’s recommendations. Family members III:4 and III:9 (Fig. 1) were screened for mutations in the known CRC-predisposing genes, *APC*, *MUTYH*, *MLH1*, *MSH2*, *MSH6* and *PMS2* using Sanger sequencing and MLPA (Multiplex Ligation-dependent Probe Amplification, MRC-Holland, Amsterdam, The Netherlands) as described in (2). Further details on primer sequences and MLPA probe kits can be obtained on request.

### Affymetrix genome-wide human SNP array 6.0

The GeneChip 6.0 platform includes about 906,600 SNP- and about 900,000 copy-number probes, covering the whole genome with an average spacing of approximately 1.3 kb. Samples were prepared according to standard conditions (Affymetrix, Santa Clara, CA). Purification of PCR products were performed using Magnetic Beads (Agencourt Bioscience Corporation, Beverly, MA). Hybridization, washing and staining of arrays were performed as described by the supplier.

### Exome capture and sequencing

DNA samples from family members III:1, III:4, IV:2 and IV:3 were quantified using the Qubit system (Life Technologies, Carlsbad, CA). DNA, were fragmented using the Covaris S2 Ultrasonicator (Covaris, Woburn, MA), where after the samples were analyzed on the Bioanalyzer (Agilent Technologies, Santa Clara, CA) for correct fragment sizes. Libraries were constructed using the Agilent SureSelectXT Human All Exon 50Mb library kit for Illumina Paired End Sequencing (v3 protocol version 1.4.1, Agilent Technologies) according to the manufacturer’s instruction. The concentration of each library was determined using the Qubit and the Bioanalyzer. Whole exome sequencing was performed on the Illumina HiScanSQ with 2×76 bp paired end reads using TruSeq SBS HS-v3 clustering and sequencing chemistry (Illumina, San Diego, CA).

### Analysis of sequencing data

Quality assessment of the sequence reads was performed by generating QC statistics with FastQC (http://www.bioinformatics.bbsrc.ac.uk/projects/fastqc). Read alignment to the reference human genome (hg19,UCSC assembly, February 2009) was done using BWA (3) with default parameters. After removal of PCR duplicates (Picard tools, http://picard.sourceforge.net) and file conversion (Samtools) (4), quality score recalibration, indel realignment and variant calling were performed with the GATK package (5). Variants were annotated with Annovar (6) using a wide range of databases such as dbSNP build 135 (7), dbNSFP (8), KEGG (9), the Gene Ontology project (10), MITOMAP (11) and tracks from the UCSC (12). The targeted region of the exome analysis included the complete coding region of the *POLE* gene. Reference sequence used for *POLE* was NM_006231.2.

### Data filtering

All common variants among the three affected individuals were filtered against one unaffected individual from the family. An in-house control data set (38 exomes of patients with different disease phenotypes), provided by Genomics Core facility (University of Gothenburg) was then applied for filtering of platform specific technical artefacts and polymorphic variants.

### Analysis of the c.1089C>A, p.Asn363Lys mutation

DNA from blood and/or paraffin-embedded tissue from family members were analyzed for the presence of the mutation using Sanger sequencing and TaqMan and the control material was analyzed using TaqMan. The mutation was confirmed on a second PCR sample. Custom TaqMan Assay Design Tool (Life Technologies) was used to design the probe. The analysis was preformed according to the manual instructions in 96-well plates on an Applied Biosystems 7900HT fast real-time PCR system (Applied Biosystems, Foster City, CA). The analyses were carried out at the Genomics Core Facility at the University of Gothenburg.

## Results

The first attempts to identify the genetic cause of the disease in the family were by screening known CRC-predisposing genes (including *APC*, *MUTYH*, *MLH1*, *MSH2*, *MSH6* and *PMS2*) by conventional Sanger sequencing and MLPA. These analyses all turned out negative and further attempts to isolate a possibly new CRC predisposing gene were done in 2007 when a linkage analysis was performed with Affymetrix GeneChip Mapping 10K Array. However, these analyses did not reveal any region of linkage and recently a re-analysis of the 10K array revealed a low SNP coverage and poor marker informativity in the *POLE* region for family members. In fact *POLE* maps distal to the most terminal chromosome 12p marker on this array (position Chr12:133,200,348–133,263,945; Genome map build GRCh37/hg19). Re-analyses of family members using an array with an approximately 100 times denser marker set (Affymetrix Genome-Wide Human SNP Array 6.0) revealed a shared haplotype between the flanking markers rs1051219 at position 132,237,750 and rs2291256 at position 133,393,323, and at least one crossover beyond the coverage of the Affymetrix 10K array which explains why linkage could not be established with the 10K array. A linkage analysis of the detected mutation using Array 6.0 data gives a maximum LOD=3.7, which is genome-wide significant. Using whole-exome sequencing performed on four individuals in the family (Fig. 1) we identified a missense mutation in the *POLE* gene, NM_006231.2:c.1089C>A, p.Asn363Lys, in affected individuals (Fig. 1). A local control material consisting of 642 healthy blood donors (i.e. 1284 alleles) from the western part of Sweden was also negative for the mutation. The mutation is not present in the NHLBI GOExome Sequencing Project (ESP) (https://esp.gs.washington.edu/drupal/) nor in dbSNP (NCBI) (http://www.ncbi.nlm.nih.gov/SNP/). The human *POLE* sequence contains a synonymous SNP (rs146639652 C/T) at the same position with a minor allele frequency in Americans of European decent of 0.0001 (based on ESP6500). The TCGA (The Cancer Genome Atlas, http://cancergenome.nih.gov/) data set was examined for possible somatic presence of the mutation in colon adenocarcinoma, endometrial tumours, serous cystadenocarcinoma and brain low grade glioma without any findings.

**Figure 2.**
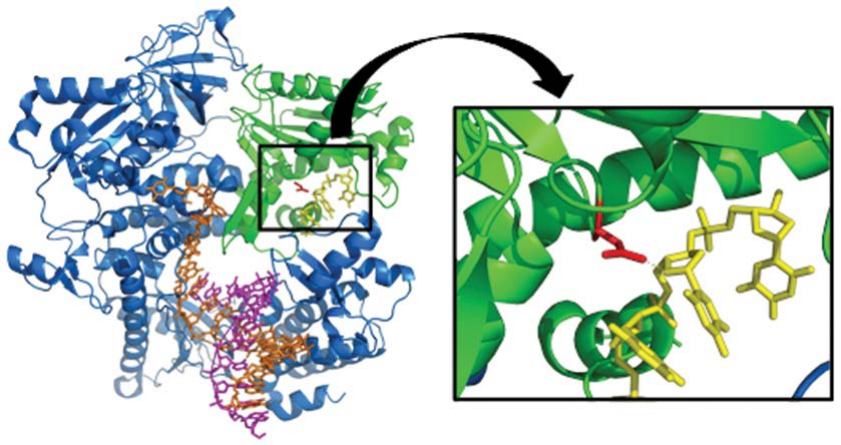
Superposition of the yeast DNA polymerase (3IAY.pdb) and ssDNA substrate from the *Escherichia coli* DNA polymerase Klenow fragment (1D8Y.pdb) with the polymerase domain (blue), the double stranded DNA (brown and magenta) and the proof-reading exonuclease domain (green). The ssDNA is shown in yellow. Magnification of the polymerase binding to an ssDNA; The NH_2_-group of the Asn side chain (red), structurally equivalent to the human *POLE* p.Asn363, forms a direct hydrogen bond with the 4’oxygen of the ssDNA backbone. PyMOL (http://www.pymol.org) was used for generating the structure superposition.

The substitution p.Asn363Lys has a profound effect on the substrate binding at the active site of the proofreading exonuclease domain. The structural homology of this domain is well conserved between species. In comparing eukaryotes and prokaryotes, the metal-coordinating active-site residues and the invariant p.Asn363 can be superimposed with a root-mean-square deviation of 0.4 Å. A high-resolution structure of the human polymerase ɛ is lacking, but the structure of the yeast *Saccharomyces cerevisiae* polymerase δ (Pol δ also known as Pol3) (3IAY.pdb, Protein Data Bank, www.rcsb.org) and the *Escherichia coli* Klenow fragment (KF) with bound ssDNA substrate (1D8Y.pdb, Fig. 2), provides, on the atomic level, understanding of the pathogenic properties of the amino acid substitution that we report. The side chain NH2-group of the Asn can form a direct hydrogen bond with the 4’ oxygen of the substrate backbone. This interaction is important for exonuclease activity (13). In the KF, the rate for excision of 3’ nucleotide residues drops up to 100-fold if the corresponding Asn (Asn420 in the KF) is substituted for Ala (14), and the exonuclease activity drops a 1,000-fold when Asn is replaced with Asp in the DNA polymerase of the thermophilic *Thermococcus kodakarensis* (15). These mutations emphasize the necessity of the precise conservation that exists in the wt enzyme. The Ala side chain is small and does not impose steric hinders. Similarly, the Asp side chain is of the same size as the wt Asn, but, neither Asp nor Ala nor any of the other amino acids (possibly with the exception of Ser) can readily form the required stabilizing hydrogen bond to the substrate.

**Figure 3.**
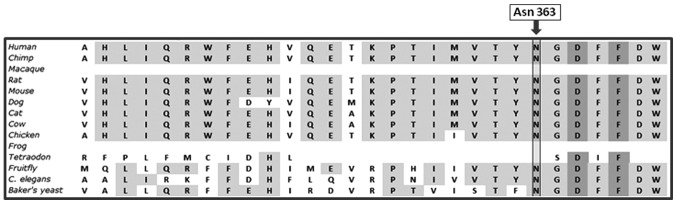
Alignment of ortholog *POLE* sequences showing conservation of the p.Asn363 amino acid between species.

The very high degree of conservation of the p.Asn363, strictly conserved in the exonuclease domain (Fig. 3), and the p.Leu424 (only replaced with Met in the KF) in the B type polymerases demonstrates their importance for enzyme functionality. The side chains of p.Tyr362 and p.Leu424 together form a platform for the single-stranded primer DNA and in *S. cerevisiae*, the Leu479Ser (corresponding to p.Leu424) mutant completely lacks exonuclease activity (16). However, when the Leu is substituted for Ala in the T4 DNA polymerase, approximately 25% exonuclease activity is retained (17), and the severity of the potentially milder p.Leu424Val substitution is difficult to assess correctly. The difference in phenotype of our family with the apparently more severe p.Asn363Lys mutation compared to the phenotype of the family with the p.Leu424Val mutation (only CRC in carriers) can probably be correlated with to what extent the activity of the exonuclease function is perturbed in the two mutant proteins.

Family members carrying the p.Asn363Lys mutation demonstrate a highly penetrant predisposition not only to CRC but also to extra-intestinal tumours such as ovarian, endometrial and brain tumours. In one case a mutation carrier also developed pancreatic cancer at age 78 (Fig. 1, family member II:9). The mutation was present in heterozygous form in 12 family members. A limited number of colorectal adenomas in the range resembling AFAP or *MUTYH*-associated polyposis were found in mutation carriers and several of them had undergone polypectomy prior to development of CRC. To investigate the incidence of *POLE* mutations in the population from the western part of Sweden (from where the family originates), 87 patients with a history of familial CRC or disease onset at young age were examined for mutations in the exonuclease domain of *POLE* (exons 3–14, codons 69–491). The patients had no colorectal adenoma or a number of adenomas in the range between a few and approximately 100. They had all been shown to be negative for mutations in either the mismatch-repair genes, *MSH2*, *MLH1*, *MSH6* and *PMS2* and/or *APC* and *MUTYH*. No mutation in *POLE* was found in any of these patients, and it was concluded that mutations in this gene only is responsible for a minor fraction of inherited CRC in our region.

Two colorectal tumours, one from each of family members III:9 and IV:2, and also a colorectal adenoma from member IV:3, were examined for second hits in *POLE* using whole-exome sequencing and loss of heterozygosity (LOH) of the wt allele was examined with microarray analyses, but no aberrations were identified. Second hits in *POLE* have been reported (Cancer Genome Atlas Network) (1,18–21) in some CRC tumours which could imply a tumour suppressor activity of the gene but the question whether *POLE* act as a classical tumour gene is not yet clear. Five colorectal tumours from three affected individuals were all microsatellite stable (MSS) which is in line with previous findings that tumours with mutations in *POLE* are MSS and are hypermutated with mutations in other genes involved in the classical tumorigenesis pathway (1,21).

## Discussion

The family presented here has been a research subject since 1980 due to the striking dominant inheritance of tumours and a clinical description of the family was published 1984 (22). A first report relating to the genetic cause of disease on chromosomal level was published in 1989 (23). During the years the family has been screened for deleterious mutations in known CRC-predisposing genes without any findings. Exome sequencing finally made it possible to identify the genetic cause of disease in the family. The mutation is likely to have a profound effect on the proofreading activity of the exonuclease function of *POLE* as is shown by mutagenesis studies performed in microorganisms and further corroborated by the high degree of conservation at this amino acid location.

It has been suggested that a possible genotype to phenotype correlation might exist in the polymerase proofreading-associated polyposis. Palles *et al* (1) reported that the mutation, p.Ser478Asn, in *POLD1*, encoding the exonuclease domain of polymerase δ predisposes carriers not only to colorectal tumours but also to endometrial cancer and possibly also to brain tumours. The apparent difference in tumour spectrum between carriers of the *POLE* p.Leu424Val and the *POLD1* p.Ser478Asn substitutions was unexplained and the authors mention that similar differences are found in Lynch syndrome where endometrial cancer seems to be more frequent in carriers of *MSH6* mutations. However, the p.Asn363Lys mutation reported on here clearly demonstrates that mutations in *POLE* can predispose carriers to a broad spectrum of tumours. When comparing the effect of the two amino acid substitutions, the p.Leu424Val reported (1) and the p.Asn363Lys presented here, it is possible to more accurately suggest that a genotype to phenotype correlation exist for this gene. The p.Asn363Lys substitution that we now report on have a more drastic effect on the proofreading activity which seemingly has the potential to initiate the development of various tumours in carriers, while the p.Leu424Val mutation seems to have a more limited effect which restricts carriers to the development of colorectal tumours. We thus suggest that a genotype to phenotype correlation needs to be considered in the clinical follow-up of *POLE* gene mutation carriers.
